# Motivators of Indiscriminate and Unsafe Supplement Use among Young Australians

**DOI:** 10.3390/ijerph18199974

**Published:** 2021-09-22

**Authors:** Alexander Campbell, Julia Carins, Sharyn Rundle-Thiele, Sameer Deshpande, Bradley Baker

**Affiliations:** 1 Social Marketing at Griffith, Griffith Business School, Griffith University, Nathan, QLD 4111, Australia; j.carins@griffith.edu.au (J.C.); s.rundle-thiele@griffith.edu.au (S.R.-T.); s.deshpande@griffith.edu.au (S.D.); 2 Defence Science & Technology Group, Land Division, Scottsdale, TAS 7260, Australia; Bradley.Baker2@dst.defence.gov.au

**Keywords:** Theory of Planned Behavior, supplements, theory, program planning

## Abstract

Background: There is growing concern about the self-administration of supplements, which can often be indiscriminate, counterproductive to health, and serve as a gateway to more harmful drugs and substances. Research suggests that high uptake of performance- and image-enhancing drugs (PIEDs) is correlated with body image to accentuate masculinity. This study provides insights into limiting unhealthy supplement usage. This research identifies reasons for casual unhealthy supplement use among young adult Australians through the Theory of Planned Behavior (TPB) lens, providing practitioners with insights into developing interventions to deter their use. Method: Semi-structured in-depth interviews were conducted with ten participants aged between 18 and 40, using a convenience sample. Leximancer analysis was used to assess word co-occurrence and map to TPB constructs. Results: Leximancer identified positive attitudes, social norms, and perceived behavioral control towards supplement usage. Key themes that influenced supplement use were weight loss, body image, nutrition, training, education, challenges, need, and time. Furthermore, using TPB constructs, affective and instrumental attitudes and prevailing norms were observed when investigating what would cause an individual to use supplements in an unhealthy manner. Conclusion: Through understanding the motivations of indiscriminate supplement use across the Australian population, the study has uncovered several social factors that may reduce or limit the practice of unsafe supplement usage.

## 1. Introduction

In Western societies, the use of supplements that pose health risks is increasing [1,2,3,4]. This may involve indiscriminate self-administration of substances that are often untested, unlikely to pass a “risk-benefit assessment”, not recommended by a health professional, banned in sport, and illegal [5]. Broadly, supplements have been defined as “a food component, nutrient, or non-food compound that is purposely ingested in addition to the habitually-consumed diet to achieve specific health and performance benefit” [6]. This definition covers a wide range, from vitamins and minerals to performance- and image-enhancing drugs (PIEDs). Commonly used PIEDs are growth hormones, peptides, and anabolic–androgenic steroids [7,8].

Australia has witnessed increasing use of PIEDs and lifetime usage rates of anabolic–androgenic steroids [9]. Moreover, users of PIEDs commonly consume many supplements simultaneously (often referred to as stacking), increasing the risks of adverse side effects [10]. There is a high prevalence of supplement usage in Australia, with vitamins, fish oils, herbal supplements, multivitamins, and multi-minerals being commonly used [11]; thus, usage could be considered as normalized within the young Australian population, defined in this study as 18 to 40 years of age.

Most users of supplements such as anabolic–androgenic steroids use them to enhance appearance [12]. Additional motivations for anabolic–androgenic steroid consumption include a desire for masculinity and dissatisfaction with body image [13,14,15]. Gender differences have been identified, where men use supplements to build muscle mass, decrease recovery time and increase overall power and strength. In contrast, women seek to overcome nutritional deficiency, enhance beauty, lose weight, and maintain energy [16,17]. For young adult athletes, supplement use is split into two predominant areas: healing/recovery and sports performance. For example, protein and creatine are used to enhance athletic performance, and vitamins and minerals are considered to assist during recovery [18]. Factors influencing an athlete’s decision to use supplements may include enhancing strength and endurance, reducing recovery from injury, and dietary intake substitution [18]. External influences include recommendations through a personal network, merchants, or the internet [19]. In summary, there is a range of reasons why people commence or continue to use supplements.

Supplement use has recently increased due to the misconception that all plant-derived ingredients pose no health risks [20]. The use of lower-risk supplements (e.g., nutritional supplements) for muscle gain by young people can represent a pathway to unhealthy supplement usage such as anabolic–androgenic steroids [21]. For athletes, legal supplements can act as a gateway to banned or illegal substances [22]. Competitive athletes that use legal supplements (e.g., nutritional supplements) versus those who do not are three and a half times more likely to engage in doping [22], which is defined as the illegitimate use of PIEDs by athletes, amateurs, or professional sportspeople for competitive advantage [23]. Significant differences in beliefs and attitudes towards supplements exist between users and non-users of supplements, indicating that legal and non-banned supplements users are more likely to engage in unsafe supplement use than non-users [22]. This indicates that understanding why users consume supplements can be informative when aiming to prevent use or practices involving health risks.

Common side effects experienced by supplement users include numbness, tremors, shaking, flushing, headaches, abdominal pain, anxiety, dizziness, mood swings, acne, and constipation [24]. There have also been reports of sports supplements causing serious health issues. For example, a young, healthy male experienced a hemorrhagic stroke after taking a sports supplement that lacked a defined disclaimer [25]; other symptoms include palpitations, chest pain, or tachycardia [26]. PIEDs’ adverse health effects include cardiotoxic events, cerebral strokes, and psychiatric symptoms [27]. In addition, anabolic–androgenic steroids have adverse effects on the cardiovascular system, atherosclerosis, hypertension, arrhythmia, thrombosis, and erythrocytosis [28]. Although there are supplements that do not pose health risks when used in the prescribed or recommended way, there are some (e.g., anabolic–androgenic steroids) that pose a serious threat to health and well-being [29] and others that pose risks when used indiscriminately (e.g., consumption of multiple supplements, leading to harmful levels of specific substances or interactions between substances) [30]. In addition to health risks, there is the risk of punitive action for PIED users, who, despite the legislative prohibition, are able to obtain PIEDs illegally in the fitness industry [31]. This emphasizes the need for more behavior change interventions to demarket risky supplement usage among identified at-risk and current user groups.

In a bid to design more effective behavioral interventions, an extended understanding of why users are attracted to supplement use is required, particularly for identified at-risk and current user groups. It has been claimed that behavior change efforts are enhanced by applying theory, as theory allows for a rich and robust understanding of how things work [32]. Willmott and Rundle-Thiele [33] synthesize theory application, explaining why and how theory should be used across planning, design, implementation and evaluation phases. Adopting a theory-based approach to guide formative research and program design will allow drivers of the focal behavior to be identified and targeted during intervention, and will support detailed evaluation of programs, leading to refinement for future iterations [34]. This study demonstrates how theory could potentially be applied in program planning and design. Specifically, this study seeks to understand what factors should be targeted to elicit the desired change in behavior (e.g., reduction in indiscriminate and unsafe supplement use).

The Theory of Planned Behavior (TPB) [35] was selected as the guiding theoretical framework for this study. The TPB has been found to explain intentions across a range of intentional, planned behaviors. For example, TPB has been used to explain binge drinking among under-aged university students [36,37]. Alcohol drinking is a deliberate and planned behavior requiring effort to access alcohol for purchase and later consumption. TPB has also demonstrated an ability to identify factors to target for change in other intentional contexts, including exercise during pregnancy [38], dietary supplement consumption among HIV-positive black women [39], cervical cancer screening [40], obesity preventions [41], and quitting of smoking, which is a highly addictive substance requiring strong, intentional effort [42].

**Figure 1:**
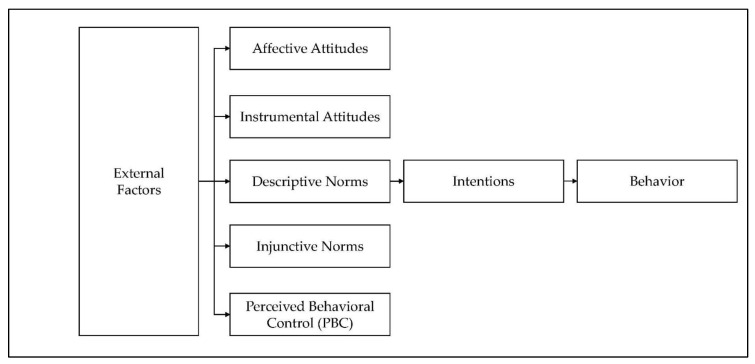
Adaption of Ajzen’s Theory of Planned Behavior (TPB). 2011.

Taken together, a review of the literature identified that many studies demonstrate the capacity for TPB to explain and predict behavior through a set of clearly defined factors. Meta-analytic studies indicate that the TPB framework can effectively predict health behavior across many behaviors, explaining 19% variation in action and 43% in intentions [43]. TPB is a popular framework with social marketers [44,45] because of its power to predict behavior through understanding attitudes, subjective norms, and perceived behavioral control. According to the TPB, three key factors contribute to intentions: attitudes towards the behavior, perceived behavioral control (PBC), and subjective norms [35], and in turn, intentions explain behavior. Figure 1 explores the two-concept TPB model [46], where:

- Affective attitude—the behavior enjoyable to the individual;
- Instrumental attitudes—the behavior beneficial to the individual;
- Descriptive norms—if other individuals conduct the same behavior;
- Injunctive norms—if others influence the behavior;
- Perceived behavioral control—the individual’s confidence in executing an action, and how those constructs can shift intentions.

With many substances banned for use in Australia, it is hypothesized that supplement use is an intentional behavior requiring cognitive effort to decide to take supplements and deliberate action to purchase substances for consumption. Given the intentionality of the behavior and evidence indicating TPB’s potential to explain intentional behaviors, the objective of this study was to apply the TPB framework to identify which theoretical constructs are involved in supplement use, and therefore, which could be targeted during the design of interventions that seek to reduce or limit indiscriminate and unsafe supplement use.

Semi-structured interviews designed using the TPB framework were used to uncover the reasons behind unhealthy supplement usage in an at-risk young adult population. Unhealthy supplement usage was introduced to participants as supplements with known health risks or practices known to increase health risks. This study demonstrates how theory can be applied to identify factors to inform intervention planning and design.

## 2. Materials and Methods

This study follows a qualitative research approach using semi-structured interviews to understand the phenomenon of unhealthy and indiscriminate supplement usage from participants’ perspectives, rather than generalizing a large dataset [47]. In qualitative data, saturation (where no new data or constructs of interest emerge) can be reached with approximately 6 to 12 interviews [48,49].

A semi-structured discussion guide was developed based on the TPB model. Table A1 in Appendix A outlines the complete discussion guide linking questions to the TPB model to provide a clear guide on how the theory was applied to identify factors to inform intervention planning and design. Furthermore, the discussion guide seeks to understand the motivations and influences of unhealthy supplement usage, where the user is consuming a level of supplements that are deemed unsafe, and indiscriminate usage, where the user is consuming supplements without careful consideration of the ingredients [29,30].

The following conditions screened participants: level of participation in physical activity, have engaged or witnessed unhealthy supplement usage, under 40 years, and reside in Australia. The recruitment of participants (n = 10) was conducted through a convenience sample method and utilized networks involved in physical activity. Participants were recruited through a social network post via LinkedIn and Facebook, direct email, and in-person recruitment. The semi-structured in-depth interviews were conducted in a 30-min timeframe, voice recorded and later transcribed for analysis. The ten interviews reached data saturation and provided suitable insights to understand the motivator of indiscriminate and unsafe supplement usage. Consent was received before the commencement of the interview, and all data were anonymized to safeguard participant privacy.

The collected data were analyzed using the word association platform, Leximancer. In the study, we used the Leximancer platform to group words, themes, and concepts via co-occurring association from the interview transcripts [50]. Leximancer was selected based on the premise that co-occurring word analysis offers statistical advantages over other qualitative techniques [51]. After the initial analysis, a thematic approach was taken to identify, analyze, and report patterns across the interview dataset [52]. The strategy of pairing the Leximancer platform with thematic content analysis was undertaken to enable the option of textual analysis [53] while remaining firm in analyzing the data objectively [54].

## 3. Results

### 3.1. Participants

Participants recruited through convenience sampling were primarily men aged from 22 to 39 years, resided in Brisbane, Queensland, Australia, and represented diverse professions (Table 1). The interviews, on average, ran for 25 min.

### 3.2. Visual Concept Map, Counts, and Probabilities

Data revealed several concepts reflecting the participants’ thoughts and opinions, with each key concept contributing to the overall narrative of response. Table 2 breaks down the concepts by word-like, count, and relevance. Any concept with less than 10% relevance was excluded from the overall results.

**Figure 2:**
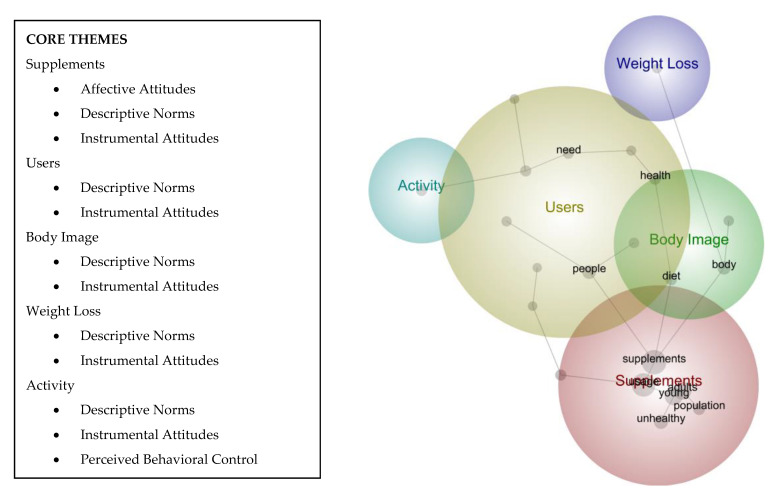
Leximancer theme map output.

The top-ranked concepts are supplements (230 counts at 100% relevance), usage (204 counts at 89% relevance), adults (90 counts at 39% relevance), young (90 counts at 39% relevance), body (82 counts at 36% relevance), people (81 counts at 35% relevance), unhealthy (51 counts at 22% relevance), gym (47 counts at 20% relevance), health (43 counts at 19% relevance), and work (37 counts at 16% relevance). Five core themes emerged from these concepts. These were supplements, users, activity, weight loss, and body image (Figure 2).

### 3.3. Interview Responses

The five key themes (supplements, users, body image, weight loss, activity) were examined in the TPB constructs of attitudes, subjective norms, and perceived behavioral control to understand how the identified themes influence behavioral intention and action.

#### 3.3.1. Supplements

The supplement theme offers the foundational layer to explore TPB concepts. Reference to supplements permitted detailed analysis of the remaining themes. Within the supplement theme, the following co-occurrences of words appeared in the participant dialogue: unhealthy (100%), population (100%), implemented (100%), usage (93%), adults (83%), young (83%), diet (80%), challenges (75%), education (58%), and training (50%) (Table 3). All respondents indicated an unhealthy trend in supplement usage across the population. In addition, respondents highlighted the challenges in education, training, and diet concerning usage levels.

These quotes further illustrate the identified themes.

*The term “unhealthy” has quite a broad meaning. To clarify that for you, and it’s your opinion of unhealthy usage instead of my opinions.*

Affective Attitudes, Male, 34 Years, Mental Health Nurse, Weightlifter

*In terms of scientific studies, there are not nearly enough peer-review scientific studies on the impact of supplements and not just the short term but also the long-term impact of supplements. They say creatine in large doses affects your liver, for example. I don’t take large doses, but theoretically, I would be interested, yes.*

Instrumental Attitudes, Male, 34 Years, Chef, Rock Climber

*My view (assumption) of supplements is if you have a balanced diet, they’re not needed. The word supplement means you’re supplementing something that’s missing from your diet, so look at your diet first.*

Instrumental Attitudes, Male, 39 Years, Small Business Owner

*I do use supplements. I use an organic protein powder during recovery days and after an extended workout. When I’m training for power, I use micro-doses of concrete creatine. It increases your overall weight, but when I’m training for power, I don’t mind having that extra weight very briefly. And I use Codrug. And then, I also use Glutamine for joint recovery because joints get fucked climbing.*

Instrumental Attitudes, Male, 34 Years, Chef, Rock Climber

*People who get interested in supplements often do research by themselves, and there is a wealth of knowledge on the internet, but you do get people who just walk into your supplement stores and grab the first thing that is suggested to them. During my creatine use, I read stories of people who used way too much of it and then ran into problems down the line.*

Descriptive Norms, Male, 33 Years, Martial Arts Instructor

Respondents indicated a lack of information available on supplements, and if supplements are harmful, they would like to know. Other respondents demonstrated awareness that supplements are not needed if their diet is balanced and delivering needs. These responses gave insight into the perspective of supplement use. The above quotes indicate the instrumental and affective attitudes of supplement usage. Moreover, respondents’ attitudes towards supplements seem to be influenced by health, diet, recovery, and nutrition, with one quote questioning the notion of what is unhealthy. Respondents also showed a clear line of questioning regarding body image and unhealthy supplement usage, contesting the evidence, being uncertain, and formulating evidence-based assumptions—thus demonstrating skepticism and the need for further research. Furthermore, instrumental and affective attitudes provide evidence that health, fitness, and nutrition influence unhealthy supplement usage.

#### 3.3.2. Users

The term “users” refers to the participants’ thoughts and opinions on supplements by other users. The probability of the sub-term being referred to in the Leximancer analysis is as follows: work (32%), protein (29%), time (27%), gym (26%), health (21%), body (21%), need (18%), nutrition (17%), and education (17%). Thus, this indicates that supplements users are influenced to reduce or increase usage based on education levels, nutrition value, body image, and recovery time. This is also substantiated by the evidence of subjective norms in the participants’ responses in injunctive and descriptive norms. When referring to the co-occurring words across sub-concepts and the primary concept, body (17), gym (12), and work (12) were all regularly used. The following quotes emphasize the respondents’ opinions about users.

*I think the number one reason why people do supplements is because of body image and how they perceive themselves, and how they compare themselves to others, because I think a lot of people who use supplementation have specific body images in mind, and they compare themselves to their peers.*

Descriptive Norms, Male, 28 Years, Scientist, Futsal Player

*People need to be realistic, and I think that it’s just that whole macho bravado across men and women, where women want to be, or they want to compete with men, and social media allows them to compare and constantly. This person’s lifting 325 so, they have to do that.*

Descriptive Norms, Male, 35 Years, General Practitioner, Weightlifter

*I know it fairly well, and my usage is fairly limited, I would say, so yes, I would benefit, potentially. I would say there’s never a bad situation to have more information about it. More information is always helpful. In terms of scientific studies, there are not nearly enough peer-review scientific studies on the impact of supplements and not just the short term but the long-term impact of supplements. They say creatine in large doses affects your liver, for example. I don’t take large doses, but theoretically, I would be interested, yes.*

Instrumental Attitudes, Male, 34 Years, Chef, Rock Climber

Respondents indicated a linkage between behavior and awareness regarding health implications with supplement use and made the correlation that people who have a poor diet are inadequately educated in nutrition. Furthermore, the respondents also indicated descriptive norms regarding the concerning competitiveness within the alpha dominant genders. In addition, descriptive norms also indicated how body image is a driver of comparison between the individual and their peers, which also links back to the competitiveness of individuals. Instrumental attitudes were also identified, corroborating the need for additional educational materials.

#### 3.3.3. Body Image

Body image is another key theme featured in the Leximancer analysis. The term aligns closely with health, weight, need, and nutrition. Sub-terms related to body image are population (35%), weight (33%), need (24%), trying (23%), people (21%), health (21%), adults (19%), young (19%), nutrition (17%), and diet (17%). Therefore, one could postulate that weight gain or loss could influence unsafe supplement usage, circumventing nutrition and healthy diets, and the need to compete with their peers, emphasizing body dysmorphia. When reviewing the co-occurring word count, body image was found to be aligned with people (17), young adults (17), and population (9).

*Of course, it does. Again, it goes back to body image. It’s about how many likes you get and how many videos or responses you’ll get at the end of the day.*

Instrumental Attitudes, Female, 32 Years, Salsa Instructor

*If it’s excess, you urinate it out, and it’s purely a waste of money. Regarding those guys who want to be professional bodybuilders or in that industry, yes, they need those supplements because there is no way for someone to eat, say, 7000 or 6000 calories to support an extra 15 kilos.*

Instrumental Attitudes, Male, 35 Years, General Practitioner, Weightlifter

*Mostly personality reason and alpha males tend to try and gain an advantage to maintain their alpha status, and they tend to be the ones to end up being gym junkies, and in my experience, the ones more likely to take your heavier supplements such as steroids and that sort of thing to try and short-cut a way to a bigger body and better image.*

Descriptive Norms, Male, 39 Years, Small Business Owner, Runner

These results further establish the theme that young adults have opinions of body image relating to health, weight, and diet. The respondents’ body image and alpha male status were drivers of supplement use, indicating affective attitudes. Other respondents reflected instrumental attitudes, stating that overdosing is a waste of money if you are not a professional athlete and that supplements are attractive for obtaining fitness, performance, and body image goals. Considering instrumental attitudes and descriptive norms indicates that the concepts of challenging alpha gender stereotypes or alpha-dominated aspirations could potentially circumvent unhealthy supplement usage.

#### 3.3.4. Weight Loss

The weight loss terms have the lowest probability of the five major themes, highlighting them as lower priorities. However, the relationship between body and weight was represented in the co-occurring word count, as it has been identified to have seven different instances within the two concepts. Leximancer uncovered the following probability of co-occurring word associations: trying (14%), training (12%), body (9%), need (6%), gym (4%), protein (4%), people (4%), health (2%), and supplements (2%). Individuals are influenced by supplements for weight loss regarding training, gym, health, and body image. The following quotes reflect upon the respondents’ opinions about weight loss.

*Certainly, young people are looking to radically change their body image. Young athletes and young people who are looking to shed weight rapidly and easily.*

Descriptive Norms, Male, 33 Years, Martial Arts Instructor

*Like I said, body image, especially if you want to go into tournaments, you’ll need something to start with, so if you’re trying to lose weight fast as well.*

Instrumental Attitudes, Female, 32 Years, Salsa Instructor

*If I had a specific goal, it would be interesting to know what the specifically indicated supplements would be that you would need to achieve that goal. For instance, when your body’s trying to lose weight, put on muscle mass, or try to improve certain fitness aspects.*

Instrumental Attitudes, Male, 28 Years, Scientist, Futsal Player

*These are generally gym people, not so many climbers. I know someone who takes eight to ten tablets a day when he’s cutting weight and a ridiculous amount of protein powders and Glutamine and creatine when he’s gaining weight. It’s ridiculous.*

Descriptive Norms, Male, 34 Years, Chef, Rock Climber

The respondents connected body image and weight loss, perceived as a personal emotional response and can incur harmful health outcomes. Moreover, respondents indicated that instrumental attitudes and descriptive norms could influence behavioral intentions to lose weight.

#### 3.3.5. Activity

Activity refers to the thoughts and actions relating to physical activity and the relationship with supplement use. It is a secondary trigger to using unhealthy supplements due to the low co-occurring word count. The probability of the co-occurring concepts between activity and sub-concepts is: implemented (50%), challenges (25%), protein (21%), work (19%), people (15%), time (13%), need (12%) and weight (10%). The co-occurring concepts indicated that work and time are potential challenges that may influence the use of unsafe supplements. The following quotes emphasize the relationship that adults under 40 years have with physical activity.

*I think young men would not like the changes at all, particularly those that actively use it. I think I would struggle with that, and young men involved in sports looking for performance enhancements.*

Instrumental Attitudes, Female, 35 Years, Retail Manager, Pole Fitness

*You could bring education into the gyms, but the gyms would need to bring it in. You can’t expect the gym to be talking about nutrition, but that is probably where it needs to be implemented.*

Perceived Behavioral Control, Male, 26 Years, Retail Worker, Body Building

*I would say it would be males over females. People that have low self-confidence or put a lot of pressure on themselves to succeed. As a result, they turn to illegal substances or unhealthy supplements.*

Instrumental Attitudes, Male, 22 Years, University Student, Local AFL

The respondents focused on education and nutrition, and emotions. Other respondents noted the instrumental attitudes of users as they are not receptive to health advice regarding supplements. This was further substantiated with the affective attitudes of users concerning the external pressures to succeed. Participants indicated low ability and, hence, low perceived behavioral control to avoid supplements when not provided with nutritional education.

### 3.4. Synthesis of Themes

The Leximancer analysis uncovered strong themes and concepts. The information gathered from the semi-structured interviews helped understand the influences that encouraged supplement usage through the lens of the TPB constructs, namely behavioral intentions, social norms, attitudes, and perceived behavioral control. Perceived behavioral control was observed to a limited extent in the themes.

The key themes that influenced supplement use were weight loss, body image, nutrition, training, education, challenges, need, and reduced recovery time. The analysis helped understand what influences a young adult to start using supplements. From a TPB perspective, affective and instrumental attitudes were observed frequently. Affective attitudes were directed to alpha gender stereotypes and their emotional reactions to unhealthy supplements, whereas instrumental attitudes were concerned with health and nutrition. At the same time, descriptive norms were central to health and education about users. The perceived behavioral control constructs were influenced by the educational, community, and fitness benefits of users.

From the themes, two underlining narratives were identified as alpha dominant and health. These narratives could inform messaging for two intervention strategies to reduce unhealthy supplement usage.

**Figure 3:**
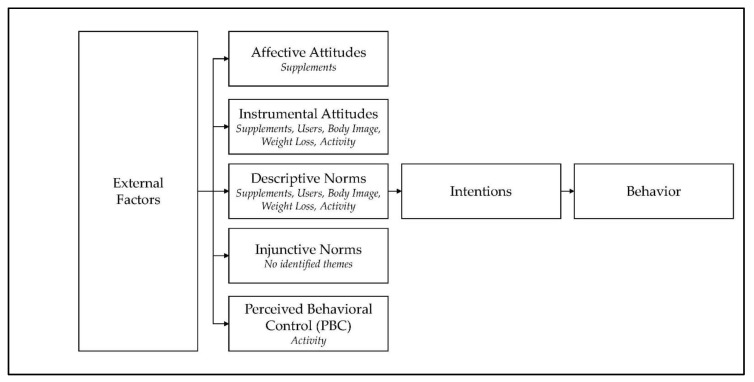
Theory of Planned Behavior with established concepts.

The majority of the TPB constructs aligned with identified themes, as illustrated in Figure 3. However, injunctive norms were not explicit. The response data could identify the individual’s feelings (affective attitude) if the behavior was beneficial (instrumental attitudes), if others conduct the same behavior (descriptive norms) and if the action of the behavior is in their control (perceived behavioral control). It was not clear whether the behavior of supplement usage was influenced by others (injunctive norms), presenting a gap within the dataset.

Other gaps were evident. The behavioral intention was present, but not well-defined in participants responses. The instrumental attitudes of participants were clear; however, affective attitudes had limited presence. Furthermore, perceived behavioral control output was also identified but limited within the dataset.

## 4. Discussion

This research aimed to identify the reasons for unhealthy supplement use among young Australians through the TPB lens to design interventions, aiming to limit, reduce, and eliminate harmful supplement usage.

Specifically, the study uncovered an individual’s motivations to use supplements linked to the desire to excel, physically dominate (an alpha-dominant aspiration) and improve health and recovery. These insights are valuable and indicate that not all users may respond to messages promoting health or identifying the risks or consequences of taking supplements due to competing desires for performance, excellence, and body dysmorphia.

This study has furthered the literature for the TPB in the health and supplement field by offering qualitative evidence that developing alpha-dominant and health-based interventions could potentially shift an individual’s motivations to use supplements in an indiscriminate and potentially unsafe manner. Whilst scholars have investigated supplements in terms of consumption, motivations, attitudes, and influences [55,56,57,58,59], this study contributes theoretical concepts that can be used to limit or reduce supplement usage within a health-focused intervention. In addition, this study contributes to both the health and social marketing literature, as the TPB constructs were used to inform potential messaging strategies to reduce or limit indiscriminate and unhealthy supplement usage. There is limited research that connects theory with a behavioral intervention, with scholars calling for more theory-led design for interventions [45,60].

Furthermore, this study corroborates a TPB supplement study by Nagar [55]. It found that attitudes affect supplement purchase intentions, and those attitudes are guided by risk–benefit, social influences, and health consciousness. Our study validates how the TPB constructs of instrumental and affective attitude, injunctive and descriptive norms, and perceived behavioral control can be used to limit or reduce unhealthy supplement usage levels through a phenomenological approach. By establishing reciprocity between interview and interviewee [61], the study uncovered a vital secondary viewpoint and perspective from participants on limiting or reducing unhealthy usage levels of supplements, giving insight on motivations and influences [62] instead of general characteristics.

In terms of method, this study used Leximancer to identify co-occurring associations within the semi-structured interviews. This allowed the analysis to theoretically link critical themes and their associations with TPB constructs, leading to a deeper understanding of behavioral intentions and influences, and better informing social marketing interventions.

### 4.1. Implication for Interventions and Program Development

This study indicates the need for a social marketing intervention highlighting the importance of diet and nutrition, supplemented with a demonstration of adverse outcomes to users of unhealthy supplements to address an individual’s attitudes towards health or physical performance.

These messages could be delivered at gyms that young people frequent via trainers and communication materials available on site. Fitness magazines, books, websites, blogs, and sports endorsements could deliver these messages. These recommendations imply the need to enhance a training curriculum that combines the dual messages of health and alpha dominance. Organizations that employ young men for their physical fitness (such as federal defense and state police) should undertake employee wellness initiatives to ensure their employees are not at risk. Finally, supplement manufacturers should push for appropriate supplements and supplement use as part of their corporate social responsibility initiatives. Potential messaging strategies could utilize affective and instrumental attitudes with the application of an alpha-dominant and health-based messaging strategy to (1) target alternative body types and challenge negative perceptions of body image, (2) define unhealthy supplement usage while highlighting the negative consequences of shortcuts, and (3) place emphasis on educational material on safe usage levels and alternative dietary options.

### 4.2. Limitations and Future Research

This study provided valuable insights into supplement use. However, some limitations must be acknowledged. These limitations offer avenues for future research. This study (n = 10) had a small sample size, which was sourced through a convenience sample. In addition, there was an uneven distribution between male and female participants, which limited the comparison between gender responses. However, the sample was sufficient as respondents’ responses reached a point of saturation, irrespective of the gender distribution. Qualitative research with small samples provides an opportunity to examine concepts in-depth and presents challenges for generalization beyond the samples achieved, while allowing for a deeper phenomenological understanding of the influences and motivations or unhealthy and indiscriminate supplement usage [46]. A larger, representative sample should be pursued to verify these findings and determine whether these insights apply to groups that differ demographically. Furthermore, future research could investigate participants who only witnessed supplement use, allowing for a greater understanding of how others perceive indiscriminate and unsafe supplements, better informing social norms. The study could have explored the TPB model constructs in more detail, specifically perceived behavioral control and subjective norms.

Leximancer’s automated analysis through statistical properties allows for identifying emerging themes via the output [63,64]. However, Leximancer has been critiqued for interpreting the data due to how the analysis is performed [65]. Compared to Leximancer, NVivo is labor intensive in the initial analysis, as the user is required to code to develop the themes or categories [54,64,66]. However, this step coaxes the researchers to be more discrete in their analysis. Therefore, using both NVivo and Leximancer in sequence would provide a more rigorous qualitative analysis through the triangulation of results and enhance understanding of the influences and motivations behind unhealthy supplement use.

Furthermore, TPB could be paired with the Social-Ecological Model (SEM) to investigate supplement use, which can help social marketers extend beyond the downstream approaches and explore potential interventions in the social and built environments [67]. SEM benefits health promotions and helps social marketers extend design thinking beyond the individuals whose behavior needs to change. SEM allows researchers to extrapolate how social influences from friends, family members, personal trainers, other gym members, and the surrounding built environment impact the behavior under study [68,69]. SEM assists when health initiatives are complex and cannot be understood from a single view [68].

Future research is needed to determine whether an intervention based on the theoretically informed insights generated in this study can effectively reduce or limit supplement use compared to a no-treatment control or an information-only intervention. There is also an opportunity to investigate if a theoretically informed social marketing intervention, such as a TPB framework, can reduce or limit unhealthy supplement usage compared to a theoretical intervention, as there are academic calls to utilize theory in intervention design and reporting [44,45,70].

In addition, future TPB studies in supplement usage could implement a longitudinal approach to address the identified gaps within the response data. Going beyond the cross-sectional dataset provided in this study will allow the TPB attitude constructs to be explored in greater detail and address the knowledge gap of injunctive norms, affective attitudes, and perceived behavioral control.

## 5. Conclusions

This study has identified factors that can be used in social marketing programs to lower the indiscriminate and unsafe supplement usage across the young Australian population. The TPB constructs of instrumental and affective attitudes delivered insight into the motivators of supplement usage. Across the synthesized themes, the reoccurring factors which influenced supplement usage were inadequate levels of education, body dysmorphia, cynicism to alternative diets, physical performance, and dietary shortcuts. Addressing these drivers of unsafe supplement usage could potentially limit supplement stacking and the use of PIEDs among the young Australian community. Furthermore, the Leximancer analysis has provided a deep analysis of core concepts and themes through linking co-occurring word-like relationships, offering statistical interpretations of TPB constructs, specifically with affective and instrumental attitudes. These results conclude that young adults have opinions of body image relating to health, weight, and diet. This can be translated to defining the critical narratives of health-based (instrumental attitudes) and alpha-dominant (affective attitudes) messaging for potential intervention strategies. Therefore, using a combination of messaging strategies based on instrumental and affective attitudes, a potential campaign could be developed to help limit or reduce unsafe supplement usage within the young Australian adult population.

## Figures and Tables

**Table 1:** Participant profile.

	Age	Gender	Profession	Supplement Use	Exercise Rate
Participant 1	22	Male	University Student	Moderate	Greater than 3 times per week
Participant 2	28	Male	Scientist	Limited	Once per week
Participant 3	39	Male	Small Business Owner	Limited	Once per week
Participant 4	35	Male	General Practitioner	Limited	Greater the three times per week
Participant 5	34	Male	Mental Health Nurse	Moderate	Twice a week
Participant 6	33	Male	Martial Arts Instructor	Previous user	Greater the three times per week
Participant 7	34	Male	Chef	Undisclosed	Greater the three times per week
Participant 8	26	Female	Retail Worker	Moderate	Greater the three times per week
Participant 9	32	Female	Retail Manager	Moderate	Twice a week
Participant 10	35	Female	Salsa Instructor	Limited	Greater the three times per week

**Table 2:** Ranked themes—semi-structured interviews.

Word-Like	Count	Relevance
Supplements	230	100%
Usage	204	89%
Adults	90	39%
Young	90	39%
Body	82	36%
People	81	35%
Unhealthy	51	22%
Gym	47	20%
Health	43	19%
Work	37	16%
Education	36	16%
Need	34	15%
Diet	30	13%
Population	26	11%
Training	26	11%
Protein	24	10%
Nutrition	23	10%
Trying	22	10%

**Table 3:** Supplement concept associations and probability.

Word-Like	Count	Relevance
unhealthy	51	100%
population	26	100%
implemented	2	100%
usage	189	93%
adults	75	83%
young	75	83%
diet	24	80%
challenges	3	75%
education	21	58%
training	13	50%

## Data Availability

The data presented in this study are available on request from the corresponding author. The data are not publicly available due to privacy and ethical reasons.

## Appendix A

**Table A1:** Unhealthy supplement usage semi-structured interview guide.

Stimuli	TPB Constructs
*Q1. Can you tell me briefly about yourself and what physical activity you undertake?*	Investigating all TPB constructs To open the conversation, establish rapport, and obtain background information about the interviewee and their relationship with physical exercise
*Q2. To begin, I would like to gain your first thoughts on a few different topics. Can you tell me what first comes to mind when I say?* • *Gym* • *Fasting* • *Healthcare* • *Nutrition* • *Cross Fit* • *Cardio* • *Weightlifting* • *Endurance* • *Aerobic* • *Balance* • *Calisthenics* • *Body Image* • *Supplement* • *Attitude* • *Rhythm*	Investigating all TPB constructs To relax the participant and create an environment that encourages them to share their thoughts and opinions openly Direct the interviewee’s attention towards the topic of interest Encourages forthrightness in answers Insights into true feelings as answers will be their first thoughts
*Q3. Regarding diet, what do you think about the use of supplements in your training regime?*	Investigating instrumental attitudes Gaining young Australians’ perspective on unhealthy supplement usage
*Q4. If using supplements, what supplements are they?*	
*Q5. Would you benefit from supplement usage education?*	Investigating perceived behavioral control Identifying emotional barriers and enablers
*Q6. What do you see as disadvantages to reducing supplement usage?*	
*Q7. What comes to mind when curbing risky supplement usage across young adults?*	Investigating affective and instrumental attitudes Identifying cognitive, actual barriers, enablers, exploring individual behavioral responses, and identifying underlying attitudes
*Q8. What challenges do you foresee if an initiative is implemented to reduce or curb risky supplement usage?*	Investigating perceived behavioral control Identifying the level of perceived control over young adult unhealthy supplement usage, and any internal or external barriers to these perceptions
*Q9. What factors would cause young adults to start or continue to use risky supplements?*	
*Q10. If you want to reduce unhealthy supplement usage levels within the young adult population, whom do you turn to?*	Investigating descriptive and injunctive norms Understanding key influencers may be determining the use of risky supplements within the young adult population.
*Q11. What is the least beneficial resource when educating young adults about unhealthy supplement usage?*	
*Q12. Within the young adult population, who do you think are using supplements for performance enhancements?*	
*Q13. Within the young adult population, who do you think are using supplements to improve body image?*	
*Q14. When it comes to educating unhealthy supplement usage within the young adult population, who do you believe would be opposed to change, and who would be receptive?*	Investigating behavioral intentions This section refers directly to opinions on supplement usage within the young adult population to ascertain their normatively held beliefs
